# High-performance human resource practices, organizational identification and employee commitment: the moderating role of organizational culture

**DOI:** 10.3389/fpsyg.2024.1494186

**Published:** 2024-10-29

**Authors:** Yifan Yang, Ahmed Mohammed Sayed Mostafa

**Affiliations:** ^1^University of Bristol Business School, University of Bristol, Bristol, United Kingdom; ^2^Leeds University Business School, University of Leeds, Leeds, United Kingdom; ^3^Faculty of Commerce, Assiut University, Assiut, Egypt

**Keywords:** high-performance human resource practices, organizational identification, employee commitment, organizational culture, social identity theory (SIT), social exchange theory (SET)

## Abstract

**Purpose:**

Drawing on social exchange and social identity theories, this study examines the mediating role of organizational identification on the relationship between high-performance human resource practices (HPHRPs) and employee commitment. The study further examines the moderating role of organizational culture in this mediated relationship.

**Method:**

A cross-sectional survey was conducted with 340 employees from state-owned enterprises in China, and SPSS was used to test the hypothesized relationships.

**Findings:**

The results indicate that organizational identification acts as a mediator of the relationship between HPHRPs and employee commitment. Additionally, organizational culture moderates the strength of this mediated relationship, affecting the degree to which HPHRPs foster commitment.

**Originality:**

This study contributes to the literature by integrating social exchange and social identity theories to explain the psychological mechanisms underlying the employee-organization relationship. It also extends the understanding of how organizational identification mediates the link between HPHRPs and commitment, and how organizational culture moderates these effects, providing a more comprehensive understanding of these interrelated dynamics in organizational settings.

## 1 Introduction

In recent decades, scholarly research on high-performance human resource practices (HPHRPs) and their impact has advanced significantly. Numerous studies have consistently affirmed the positive influence of HPHRPs on organizational performance, highlighting their critical role in enhancing productivity and effectiveness within the workplace (Delaney and Huselid, 1996; Shin and Konrad, 2017; Sheng, 2022). However, some scholars (e.g., Grant and Shields, 2002; Macky and Boxall, 2007) have criticized the field’s predominant focus on “organizational outputs,” arguing that it often overlooks employees’ responses to HPHRPs. Consequently, there has been a paradigm shift in high-performance management practices research from an employer-centric approach, which emphasizes production management and views employees as costs, to an employee-centric approach, which prioritizes employee relations and regards employees as valuable resources (Noopur and Dhar, 2020).

Employee-oriented human resource practices aim to enhance organizational performance by improving the relationship between employees and the organization. These practices manifest in various forms, including commitment human resource practices, supportive human resource practices (Allen et al., 2003), and developmental human resource practices (Kuvaas, 2008). The objectives of these practices are to fortify employees’ allegiance to the organization, bolster their perception of organizational support, and foster enduring relationships with employees. These goals are integral to developing a cohesive and motivated workforce aligned with the organization’s strategic objectives (Kinnie et al., 2005; Rathnaweera, 2010).

As the focus of organizational competition shifts from tangible assets to human resources, the relationship between employees and organizations has evolved beyond mere contractual terms to become central to sustainable organizational development (Manuti and Giancaspro, 2019). This has underscored the importance of employee commitment, an attitude that reflects the relationship between an organization and an employee, and emphasizes the value of nurturing deep, loyalty-building connections that align with long-term organizational goals (Allen and Meyer, 1996). Employee commitment is a pivotal factor influencing organizational performance and overall success and one of its main predictors that has been identified in past studies is HPHRPs (e.g., Kehoe and Wright, 2013; Mostafa et al., 2015). However, in spite of the substantial body of research supporting the positive association between HPHRPs and employee commitment, the mediators and moderators of this relationship are still “poorly understood” (Mostafa et al., 2015, p.747).

This paper seeks to address this research gap and provide an understanding of the dynamics of employee-organization relationships by examining first the mediating role of organizational identification on the association between HPHRPs and employee commitment. Organizational identification refers to employees’ sense of belonging or their alignment with organizational values and goals. This alignment is manifested not only in psychological perceptions but also in the daily behaviors of employees (Ashforth and Mael, 1989). Drawing on social exchange theory (SET; Blau, 1964), the study highlights how employee commitment is shaped through the reciprocal exchange between the organization and its employees. The study proposes that employee perceptions of HPHRPs, as part of this exchange, will foster a sense of obligation to reciprocate through higher commitment. On the other hand, social identity theory (SIT; Tajfel, 1982) is used to explain how employees’ sense of belonging and identification with the organization—derived from their self-concept and affiliation with their employer—plays a mediating role in the relationship between HPHRPs and commitment. Through this lens, organizational identification is viewed as a crucial factor in understanding how employees internalize organizational values and practices, leading to deeper commitment.

Furthermore, the study explores the moderating role of organizational culture on the relationship between HPHRPs, organizational identification and employee commitment. Organizational culture, characterized by the shared values and beliefs within an organization, significantly influences employees’ behaviors and decision-making processes. It acts as a critical regulatory factor in shaping the interactions between employees and their employers, underscoring its importance in organizational dynamics (Huey Yiing and Kamarul Zaman Bin Ahmad, 2009). The study proposes that organizational culture influences how employees interpret HPHRPs, which in turn affects their level of organizational identification and subsequent commitment.

This research makes three main contributions. First, by integrating SET and SIT, the study enhances the understanding of the psychological relations between individuals and their organizations. Second, by testing organizational identification as a mediator, the study advances the understanding of how HPHRPs relate to employee commitment. Finally, by examining organizational culture as a moderator, the study provides better understanding of when HPHRPs relate to organization identification and consequently employee commitment.

## 2 Theoretical framework

HPHRPs can be defined as the array of policies, strategies, and activities employed by organizations to effectively manage their workforce. Generally, these practices are designed to attract, retain, motivate, and develop employees, thereby enhancing their productivity, job satisfaction, and overall contributions to the achievement of organizational goals (e.g., Huselid, 1995; Lado and Wilson, 1994; Pfeffer, 1994; Breaugh and Starke, 2000).

Initial research in the field of HR primarily concentrated on evaluating the impact of individual human resource practices (HRPs) on organizational performance (e.g., Breaugh and Starke, 2000; Gueutal et al., 2005; Ployhart, 2006). For example, several scholars have examined recruitment practices in terms of their impact on organizational performance (e.g., Breaugh and Starke, 2000; Ployhart, 2006). Effective recruitment practices are critical for securing employees who possess the necessary skills, knowledge, and attributes for job success (Ployhart, 2006). Investing in a robust recruitment process can significantly aid organizations in attracting and retaining top-tier employees who possess the skills and qualities necessary to advance their objectives. Conversely, the adoption of subpar hiring practices can engender mismatches between employees and their respective roles, consequently precipitating a decline in overall performance (Gueutal et al., 2005).

Several scholars have also scrutinized the influence of training, and studies found that training practices exhibit a positive correlation with variables such as productivity, motivation, satisfaction, as well as employee morale (Noe and Kodwani, 2018; Singh and Mohanty, 2010; Ozkeser, 2019). Employees who undergo continuous training are better equipped to stay informed about industry trends, thereby fostering the creation of novel products, services, and procedures (Sung and Choi, 2014). Other scholars have also investigated the impact of compensation practices. Studies have shown that employees tend to exert greater effort and commitment when they perceive a direct connection between their performance and compensation (Alamelu et al., 2015; Riana and Wirasedana, 2016).

Despite the significant positive effects of individual HRPs on enhancing organizational performance, scholars argue that the influence of single practices on organizational performance is constrained (Wright et al., 1994) and a comprehensive integration of HRPs can afford an organization a sustainable competitive advantage (Becker and Huselid, 1998). As a result, in recent studies, there has been a decline in the focus on individual practices, and a greater focus on how HR functions such as recruitment, training, performance management, and compensation are integrated into overall management practices or systems.

### 2.1 HPHRPs and employee commitment: the mediating role of organizational identification

Becker (1960) initially introduced the concept of commitment, defining it as employees’ inclination to voluntarily invest in the organization. Within a specific organization, this unilateral investment encompasses all valuable assets that employees possess and can only be utilized within that organization. The greater the extent of employees’ unilateral investment, the more inclined they are to remain with the organization indefinitely. Buchanan (1974) argued that commitment not only reflects the economic bond between employees and the organization but also encompasses employees’ identification with and sense of belonging to the organization. It transcends mere economic utility as suggested by Becker (1960). Mowday et al. (1979) identified three components of commitment: acceptance of the organization’s goals and values, increased involvement in the organization, and the desire to maintain organizational membership. Wiener (1982) emphasized that employees’ commitment to the organization stems from their sense of responsibility and obligation, influenced by social ethics or group norms. Meyer and Allen (1991) proposed three dimensions of employee commitment: affective commitment, continuance commitment, and normative commitment. Meyer and Allen (1991) argued that affective commitment manifests in employees’ identification with and acceptance of organizational goals and values, reflecting the alignment between individuals and the organization. Continuance commitment signifies individuals’ awareness of the accrued potential costs resulting from the ongoing investment of their time and energy, leading them to perceive an increasing sunk cost and thereby choosing to remain as organizational members. Normative commitment represents a sense of obligation whereby employees opt to stay in the organization out of social responsibility. Some researchers have contended that the affective dimension entails a subjective evaluation of organizational attitudes, while the continuance and normative dimensions pertain to behavioral aspects that can effectively predict employees’ turnover behavior (Allen and Meyer, 1996).

Meyer and Allen (1997) revealed that organizational commitment is deeply influenced by how organizations treat their employees. SET (Blau, 1964) sheds light on how HPHRPs can significantly boost employees’ organizational commitment (Marescaux et al., 2013). SET is based on the principle of reciprocity, which assumes that individuals have the obligation to repay those who give them (Gouldner, 1960). When an organization treats employees in a positive way and provides them with economic or social emotional resources, it will initiate social exchange (Gould-Williams, 2007). Employees exchange individual labor for the remuneration of the organization, and loyalty to the organization for the care and support the organization provides. On the other hand, through the hard work of employees, the organization attains greater development. The formation of interdependence between employees and organizations is the formation of a social exchange relationship (Rhoades and Eisenberger, 2002). Unlike economic exchanges, which are based on clearly defined contractual obligations, social exchanges are characterized by trust and long-term reciprocity (Shore et al., 2006).

HPHRPs signal the organization’s willingness to invest in employees’ well-being, development and future prospects, thereby fostering a social exchange relationship rather than a transactional, short-term economic exchange (Kehoe and Wright, 2013). When employees perceive that their organization is genuinely committed to their well-being and development through the implementation of HPHRPs, they are likely to experience a sense of belonging and identify more strongly with the organization. This identification then is likely to mediate the relationship between HPHRPs and employee commitment, aligning with the principles of social exchange. In examining the relationship between HPHRPs and employee commitment, studies have explored potential mediating variables such as job satisfaction (Mahmood et al., 2019) and organizational support (Kerdpitak and Jermsittiparsert, 2020). However, there remains a scarcity of research investigating organizational identification as a mediator between HPHRPs and employee commitment. The following paragraphs shed light on the concept of organizational identification and explain how HPHRPs and identification are related.

Organizational identification is a concept rooted in SIT, and entails the cognitive process through which individuals establish a sense of belonging and membership within an organization (Van Knippenberg and Hogg, 2003). Tajfel (1978) defined organizational identification as an individual’s self-perception resulting from their affiliation with an organization, demonstrated through shared values and a sense of belonging. As organizational competition factors have evolved from physical to human resource factors, the relationship between employees and organizations has transcended mere contractual agreements to become a pivotal determinant of organizational sustainability. Organizational identification serves as a primary mechanism for promoting cohesion and solidarity within the workplace, positioning it as a critical driver of organizational success (Mael and Tetrick, 1992; Smidts et al., 2001). According to Ellemers et al. (2014), cultivating a strong sense of identification may lead to increased employee engagement and loyalty, potentially contributing to improved performance and overall organizational effectiveness. As such, understanding and leveraging organizational identification is essential for organizations aiming to thrive in today’s competitive environment.

Numerous scholars have investigated the factors influencing organizational identification. For example, Fuller et al. (2006) found that employees’ organizational identification is influenced by their perception of the organization’s reputation and internal respect. Ellemers et al. (2004) emphasize that leadership plays a crucial role in fostering organizational identification. When leaders promote a shared sense of identity within the organization, employees are more likely to align their personal goals with organizational objectives, ultimately boosting motivation. Procedural justice within an organization was found also to significantly impact employees’ organizational identification (Tyler and Blader, 2003; Gillet et al., 2013). Edwards and Peccei (2010) also found that higher levels of perceived support lead to stronger identification with the organization. In addition, Wang et al. (2017) found that corporate social responsibility (CSR) is related to organizational identification. Pagliaro et al. (2018) also found that different ethical climates are related to employee behaviors through organizational identification. Moreover, using scenario-based experiments, Teresi et al. (2019) found that an ethical climate of friendship is indirectly related to organizational commitment through organizational identification. Despite all these studies, there is a paucity of research on organizational identification within the context of HPHRPs. This paper aims to analyze the relationship between HPHRPs and organizational identification using SIT.

According to Turner et al.’s (1979) SIT, employees’ identification is a self-defined response set within the specific relationship between themselves and the group. The experiences and information acquired by employees within the organization serve as a benchmark for self-definition and the criteria for determining their inclusion within the organization (Chih and Lin, 2019). HPHRPs can address the needs of employees’ identification by fostering a positive work environment, offering fair treatment, and providing developmental opportunities (Hameed et al., 2022). Tyler and Blader (2003) posited that organizational identification is rooted in an individual’s assessment of their position within the organization. Sen and Bhattacharya (2001) also argued that people are likely to identify with an organization when they perceive their identification as enduring, distinctive, and capable of bolstering their self-esteem.

HPHRPs that acknowledge employees’ contributions and offer opportunities for advancement and development can bolster employees’ sense of self-esteem and value within the organizational context. Also, HPHRPs aimed at cultivating a positive and supportive work environment contribute to fostering favorable social comparisons, thereby strengthening employees’ identification with the organization (Liu et al., 2022). This means that the use of HPHRPs does not only increase reciprocity, but also enhances employees’ self-esteem and sense of self-worth because of organizational membership. This, in turn, will lead to increased organizational identification. Accordingly, this paper proposes the following hypothesis:

*Hypothesis 1:* HPHRPs relate positively to organizational identification.

While there are clear similarities between organizational identification and employee commitment, these two concepts are largely distinct from each other (Stinglhamber et al., 2015). Theoretical (Ashforth and Mael, 1989; Van Dick, 2004) and empirical (Van Knippenberg and Sleebos, 2006) evidence supports the differentiation between organizational identification and employee commitment. Organizational identification is associated with self-reference, which is not typically included in commitment scales. Identification predicts behaviors and attitudes based on self-categorization, while commitment predicts behaviors and other attitudes based on the quality of the social exchange relationship (Van Knippenberg and Sleebos, 2006). SIT posits that individuals derive a significant part of their self-concept from group memberships, influencing their behavior and attitudes toward organizational commitment (Ellemers and Haslam, 2012).

Various theoretical perspectives have been proposed regarding the relationship between organizational identification and employee commitment. Ashforth and Mael (1989) argue that identification can bolster support and commitment to the organization. Meyer et al. (2004) also suggested that organizational identification nurtures employee commitment toward the organization. Similarly, Marique and Stinglhamber (2011) found that organizational identification predicts employee commitment. Building on the above analysis, this paper proposes the following hypotheses:

*Hypothesis 2a:* Employees’ organizational identification relates positively to affective commitment.

*Hypothesis 2b:* Employees’ organizational identification relates positively to continuance commitment.

*Hypothesis 2c:* Employees’ organizational identification relates positively to normative commitment.

Based on the above discussion and the previously proposed hypotheses, this study posits that, based on the principles of social exchange, when employees perceive the organization’s regard and care for its workforce through the implementation of HPHRPs, they are more likely to identify with and consequently commit to the organization.

*Hypothesis 3a:* The positive relationship between HPHRPs and affective commitment is mediated by organizational identification.

*Hypothesis 3b:* The positive relationship between HPHRPs and continuance commitment is mediated by organizational identification.

*Hypothesis 3c:* The positive relationship between HPHRPs and normative commitment is mediated by organizational identification.

### 2.2 The moderating role of organizational culture

Hofstede (1998) asserted that organizational culture embodies an organization’s collective mindset and mode of operation. Organizational culture is defined as a set of shared characteristics among organizational members, encompassing beliefs, values, and behaviors (Lawson and Ventriss, 1992). It covers vitality, creativity, and entrepreneurship to ensure the organization’s long-term success (Quinn, 2006). Barney (1986) contends that organizational culture serves as a source of sustainable competitive advantage. It significantly influences the relationship between employees and organizations (Meng and Berger, 2019). Employees with a favorable perception of organizational culture are more likely to identify with the organization and remain committed to it (Schrodt, 2002).

Previous studies have primarily viewed organizational culture as a moderator in relationships involving leadership behavior (e.g., Huey Yiing and Kamarul Zaman Bin Ahmad, 2009). However, few scholars have considered organizational culture as a moderator between HPHRPs and organizational identification. This study proposes that the motivational and fairness aspects of organizational culture can significantly amplify the impact of HPHRPs. For instance, when training and reward systems are implemented within a culture that prioritizes fairness and employee development, employees are more likely to see these practices as genuine efforts to support their growth and well-being (Era, 2024). This organizational environment is highly valued by employees. When employees perceive fairness and motivational aspects within the organizational culture, they are more inclined to engage with and positively perceive organizational practices. This active engagement supports the internalization of the organization’s values and goals into employees’ personal beliefs, contributing to their identification with the organizational (Yue et al., 2021). This heightened identification, associated with a supportive organizational culture, may relate to employee commitment, as employees feel a deeper connection to the organization and a greater sense of loyalty (Sarhan et al., 2020). In light of these insights, this paper posits the following hypotheses:

*Hypothesis 4:* Organizational culture moderates the relationship between HPHRPs and organizational identification, such that the positive relationship between HPHRPs and identification will be stronger when perceptions of organizational culture are high compared to low.

*Hypothesis 5a:* The mediation effect of organizational identification on the relationship between HPHRPs and affective commitment is moderated by organizational culture, such that the mediated relationship will be stronger under high than low perceptions of culture.

*Hypothesis 5b:* The mediation effect of organizational identification on the relationship between HPHRPs and continuance commitment is moderated by organizational culture, such that the mediated relationship will be stronger under high than low perceptions of culture.

*Hypothesis 5c:* The mediation effect of organizational identification on the relationship between HPHRPs and normative commitment is moderated by organizational culture, such that the mediated relationship will be stronger under high than low perceptions of culture.

**Figure 1:**
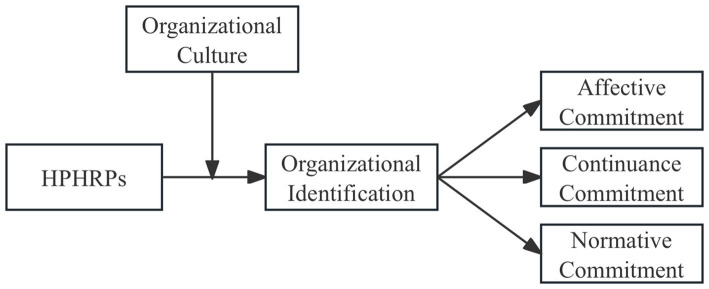
The conceptual model.

Figure 1 outlines our conceptual model.

## 3 Methods

### 3.1 Sampling

This study employed a cross-sectional design. Initially, a random sample of 400 employees from four state-owned enterprises in China, spanning the sectors of energy, transportation, manufacturing, and telecommunications, were contacted to participate in the study. These companies represent a diverse range of organizational structures and work environments, with a focus on both industrial and service-oriented operations. The participants mainly consisted of middle-level and grassroots employees who were integral to the daily operations of their respective organizations. Employees from departments such as human resources, production, sales, and customer service were included to ensure a representative sample across various functional areas. Participants responded to an online survey questionnaire via mobile phones or laptops within 7 days. Employees evaluated the organization’s HPHRPs, organizational culture, as well as their own identification and commitment to the organization. Each questionnaire was accompanied by an informed consent form, informing participants that participation is entirely voluntary, and guaranteeing that all responses collected will be kept anonymous and confidential. In total, 340 employees completed the questionnaire. Forty-five percent of these employees were female. As regards age, 36% of the respondents were between 20 and 30, 31% were between 31 and 40, 20% were between 41 and 50, while the rest were over 50. To verify the adequacy of the sample size, a *post hoc* power analysis was performed using G*Power 3.1. For the primary relationship (HPHRPs → OI), with a sample size of 400, an *α* level of 0.05, and an effect size of f^2^ = 0.11, the achieved power was 0.99. Similarly, for the moderation effect (HPHRPs x OC → OI), with the same sample size, α level, and an effect size of f^2^ = 0.05, the achieved power was also 0.99. This demonstrates that the sample size was sufficient to detect medium to large effects.

### 3.2 Measures

All variables were measured using a seven-point Likert scale, ranging from ‘strongly disagree’ to ‘strongly agree’. Additionally, the questionnaire included questions regarding the demographic information of the participants.

High-performance human resource practices were assessed using the scale developed by Mostafa et al. (2015). This scale evaluates five aspects of HPHRPS that are in line with SET and SIT: training and development, promotion, job security, communication and autonomous work design. These are ‘soft’ HRM practices that are mainly designed to create a long-term bond between an individual and the organization, and enhance employee identification and commitment (Mostafa et al., 2015; Mostafa et al., 2019; Mostafa et al., 2023). Sample items include “My organization offers opportunities for training and development” and “My organization allows me to plan how I do my work.” The reliability of the scale was good (*α* = 0.846).

Organizational identification was assessed by the scale developed by Mael and Ashforth (1992). The scale assesses employees’ identification with their organization, their sense of belonging, and their emotional attachment to it. Sample items include ‘This organization’s successes are my successes.’ and ‘I am very interested in what others think about my organizations’. The scale demonstrated good reliability (α = 0.857).

Employee commitment was measured using the scale developed by Meyer et al. (1993). The scale distinguishes between affective commitment, continuance commitment, and normative commitment. Sample items are ‘I really feel as if this organization’s problems are my own’ (affective commitment; α = 0.819), ‘If I had not already put so much of myself into this organization, I might consider working elsewhere’ (continuance commitment; α = 0.823), and ‘I feel an obligation to remain with my current employer’ (normative commitment; α = 0.811).

Organizational culture was measured using the Organizational Culture Survey (OCS) developed by Glaser et al.’s (1987). While other typologies such as Cameron and Quinn’s Competing Values Framework (1999) and Denison’s culture model offer valuable perspectives, the OCS was selected for its specific focus on practical, day-to-day organizational processes, which are more directly related to employee experiences and responses. The OCS prompts employees to view the organization as an independent entity and assess its culture across six distinct dimensions. The dimensions are meetings, information flow, teamwork, involvement, morale, and supervision. These are viewed as essential components to any organizational culture (Schrodt, 2002). They also align with the study’s focus on employee identification and commitment. In this study, these dimensions are analyzed collectively, since focusing on individual dimensions in isolation may not capture the full complexity of organizational culture. Sample items include ‘People I work with are direct and honest with each other’ and ‘People I work with function as a team’ (α = 0.873).

## 4 Analysis

First, using AMOS 23, a confirmatory factor analysis (CFA) was performed to evaluate construct validity. Model fit was acceptable (χ2(df = 362) = 957.04, *p* < 0.01; CFI = 0.88 RMSEA = 0.070 and TLI = 0.862). To ensure discriminant validity of the measures, the square root of the average variance extracted (AVE) for all constructs was calculated and compared to their respective correlations, following the methodology of Fornell and Larcker (1981). As presented in Table 1, all constructs exhibited high internal consistency with composite reliability scores above 0.75 and average variance extracted (AVE) scores above 0.50. The findings indicated that, for all constructs, the square root of the AVE exceeded the corresponding inter-construct correlation estimate.

**Table 1:** Correlations and descriptive statistics.

	1	2	3	4	5	6	7	8	9
1. Gender	—								
2. Age	0.083	—							
3. Education	−0.053	0.247**	—						
4. HPHRPs	−0.064	−0.093	−0.153**	0.712(0.861)					
5. OI	−0.047	−0.092	−0.107*	0.413**	0.738(0.857)				
6. AC	−0.098	−0.051	−0.170**	0.455**	0.420**	0.729(0.818)			
7. CC	−0.092	−0.079	−0.136*	0.638**	0.344**	0.357**	0.733(0.822)		
8. NC	−0.058	−0.101	−0.177**	0.603**	0.450**	0.504**	0.391**	0.720(0.811)	
9. OC	0.009	−0.004	−0.033	0.296**	0.304**	0.163**	0.116**	0.272**	0.732(0.784)
Mean	1.45	2.06	1.71	4.07	4.05	4.01	3.94	3.88	3.95
SD	0.498	1.001	1.085	1.000	1.441	1.473	1.491	1.509	0.997

The first entry on the diagonal is the average variance extracted square root (AVE) and the second entry (in parentheses) is the composite reliability (CR) score. HPHRPs, high performance human resource practices; OI, organizational identification; AC, affective commitment; CC, continuance commitment; NC, normative commitment; OC, organizational culture. **p < 0.01; *p < 0.05.

Then, the PROCESS macro of SPSS was used to test the proposed moderated mediation model with 95% confidence interval based on 5,000 bootstrap samples. Table 2 presents the results of testing the relationship between HPHRPs and OI. After controlling for gender, age, and education, the results indicated a significant relationship between HPHRPs and organizational identification (OI). The path coefficient was positive (*β* = 0.332, *p* < 0.01, 95%CI [0.299, 0.435]) not crossing zero, thereby supporting Hypothesis 1.

**Table 2:** Results of regression analysis (direct relationships of HPHRPs, OI, AC, CC, and NC).

	AC			CC			NC		
	β	SE	*p*	β	SE	*p*	β	SE	*p*
Gender	−0.216	0.138	0.119	−0.157	0.126	0.215	−0.057	0.128	0.658
Age	0.052	0.070	0.458	−0.003	0.064	0.958	−0.020	0.065	0.757
Education	−0.141*	0.066	0.032	−0.052	0.060	0.384	−0.104	0.061	0.088
HPHRPs	0.348**	0.055	<0.001	0.642**	0.050	<0.001	0.540**	0.051	<0.001
OI	0.281**	0.052	<0.001	0.096*	0.048	0.044	0.249**	0.048	<0.001
R^2^	0.535*			0.647*			0.644*		

HPHRPs, high-performance human resource practices; OI, organizational identification; AC, affective commitment; CC, continuance commitment; NC, normative commitment. **p < 0.01; *p < 0.05.

As shown in Table 2 also, the results revealed a significant positive relationship between organizational identification (OI) and affective commitment (AC), with a path coefficient of β = 0.281 (*p* < 0.01), thereby confirming Hypothesis 2a. Similarly, a positive significant relationship was found between OI and continuance commitment (CC) (*β* = 0.096, *p* < 0.05), supporting Hypothesis 2b. Additionally, the relationship between OI and normative commitment (NC) was also significant and positive (*β* = 0.249, *p* < 0.01), validating Hypothesis 2c. Furthermore, the analysis demonstrated significant direct relationships between HPHRPs and the three dimensions of commitment: specifically, AC (β = 0.348, SE = 0.055, *p* < 0.01), CC (β = 0.642, SE = 0.050, *p* < 0.01), and NC (β = 0.540, SE = 0.051, *p* < 0.01). These findings underscore the direct impact of HPHRPs on each commitment component within the proposed model.

The indirect relationship between HPHRPs and affective commitment (AC), continuance commitment (CC), and normative commitment (NC) via organizational identification are shown in Table 3. The indirect relationship between HPHRPs and AC was significant [*β* = 0.093, SE = 0.26, 95% CI (0.048, 0.150)], confirming Hypothesis 3a. However, the indirect relationship with CC was not significant (β = 0.032, SE = 0.019, 95% CI = −0.001 to 0.074), indicating that Hypothesis 3b was not supported. The examination of the indirect relationship between HPHRPs and NC revealed that this relationship was significant [β = 0.1083, SE = 0.025, 95% CI (0.040, 0.135)], thereby supporting Hypothesis 3c.

**Table 3:** The results for OC as a mediator of the HPHRPs-commitment relationship.

	β	SE	LLCI	ULCI
Indirect relationship: HPHRPs → OI → AC				
−1SD (2.905)	0.015	0.029	−0.045	0.071
M (4.241)	0.093	0.026	0.048	0.150
+1SD (5.576)	0.172	0.043	0.094	0.261
Indirect relationship: HPHRPs → OI → CC				
−1SD (2.905)	0.005	0.011	−0.016	0.076
M (4.241)	0.032	0.019	−0.001	0.074
+1SD (5.576)	0.059	0.034	−0.002	0.130
Indirect relationship: HPHRPs → OI → NC				
−1SD (2.905)	0.013	0.025	−0.037	0.065
M (4.241)	0.083	0.025	0.040	0.135
+1SD (5.576)	0.152	0.039	0.079	0.235

HPHRPs, high-performance human resource practices; OI, organizational identification; AC, affective commitment; CC, continuance commitment; NC, normative commitment; OC, organizational culture.

**Figure 2:**
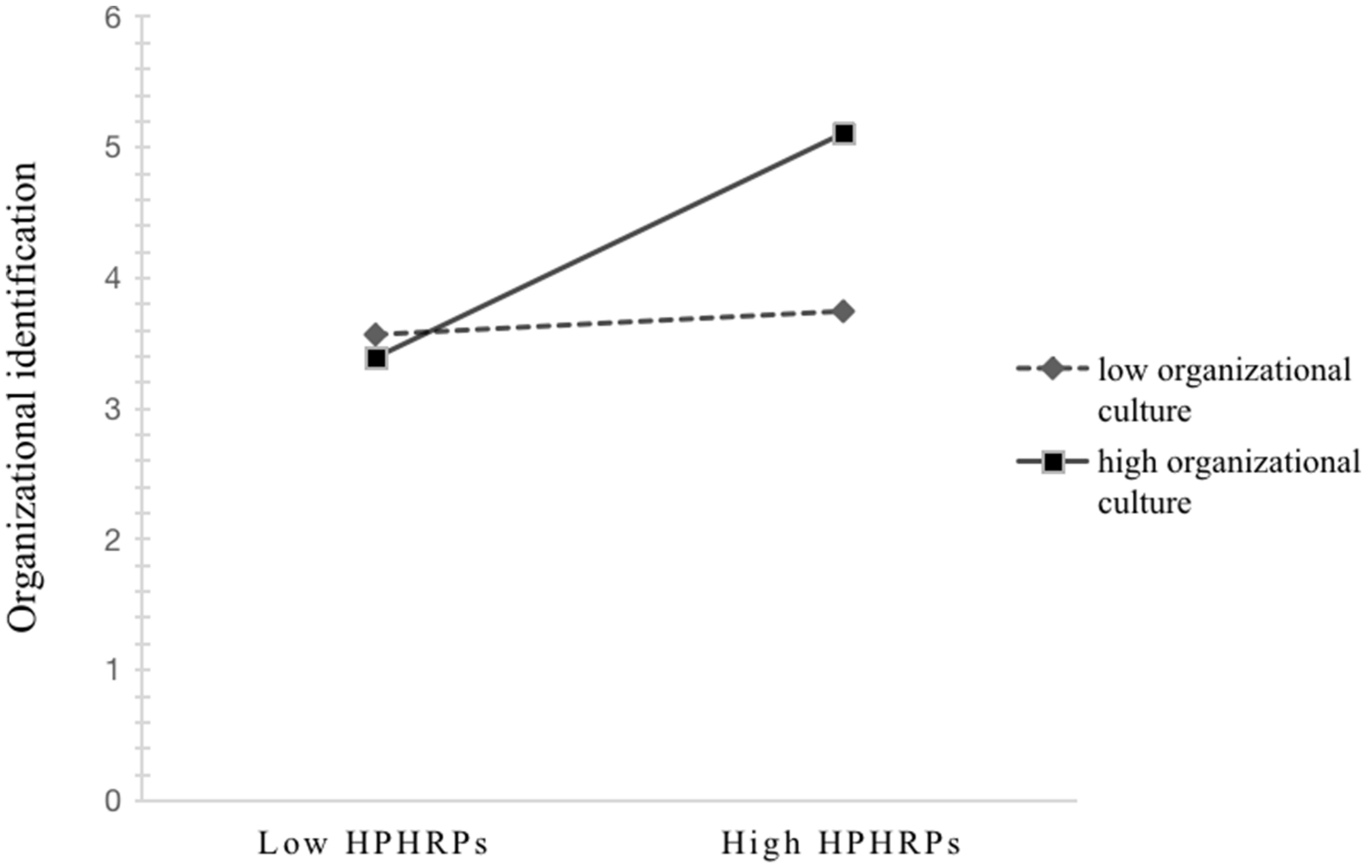
The moderating role of organizational culture in the relationship between HPHRPs and OI.

Hypothesis 4 suggests that organizational culture moderates the relationship between HPHRPs and OI. The regression coefficient of the multiplicative term (HPHRPs×OC) was significant and positive (β = 0.209, *p* < 0.001 95%CI [0.135, 0.283]), which shows that as organizational culture increased, the influence of HPHRPs on organizational identification increased. In order to more clearly reflect the moderating effect of organizational culture on the relationship between HPHRPs and organizational identification, the interaction was depicted (see Figure 2) based on the steps provided by Aiken et al. (1991). The simple slopes test revealed that the strength of the relationship between HPHRPs and OI was stronger when OC was higher (β = 0.611, *t* = 8.872, *p* < 0.01) rather than lower (β = 0.053, *t* = 0.700, *p* > 0.1). These results provide evidence to support Hypothesis 4.

As regards the moderated mediation, the conditional effects of the mediator varied at different levels of organizational culture (−1 SD as Low: 2.905; +1 SD as High: 5.576). The examination of the conditional effect of HPHRPs on AC at low (−1 SD) and high (+1 SD) organizational culture (OC) revealed that this effect was significant for high OC [β = 0.172, SE = 0.43, 95% CI (0.094, 0.261)]. The index of AC was 0.059, with a 95% bootstrap confidence interval (CI) that did not include zero (LLCI = 0.026, ULCI = 0.101), indicating a significant moderated mediation effect. Additionally, the examination of the conditional effect of HPHRPs on NC at low and high OC revealed that this effect was significant for high OC [β = 0.152, SE = 0.039, 95% CI (0.079, 0.235)]. The index of NC was 0.052 and a 95% bootstrap CI did not include zero (LLCI = 0.024, ULCI = 0.089). These results confirm Hypothesis 5a and 5c, indicating that organizational culture moderates the indirect effect of HPHRPs on affective and normative commitment. However, the indirect effect was not significant between low (β = 0.005, 95% CI = −0.016 to 0.076) and high OC (β = 0.059, 95% CI = −0.002 to 0.130) for CC. The index of CC was 0.020, as the 95% bootstrap CI included zero (LLCI = −0.001, ULCI = 0.047). This finding does not support Hypothesis 5b, suggesting that organizational culture does not significantly moderate the relationship between HPHRPs and continuance commitment.

## 5 Discussion

The empirical findings of this study corroborate prior scholarly discourse on the relationship between HPHRPs and organizational identification, as substantiated by works such as those by Hameed et al. (2022) and Chih and Lin (2019). As anticipated, a favorable association between HPHRPs and organizational identification was observed. The empirical findings of this study also confirm previous scholarly research on the nexus between organizational identification and employee commitment, as documented by Stinglhamber et al. (2015). Organizational identification had a positive relationship with the three dimensions of employee commitment: affective, continuance, and normative. This suggests that increased organizational identification fosters a deeper commitment to the organization.

The findings highlight the significant indirect impact of HPHRPs on both affective commitment and normative commitment through organizational identification. In particular, organizational identification partially mediated this relationship. Thus, when HPHRPs are effectively implemented, they foster a sense of belonging and pride among employees, which may have a relationship with their identification with the organization. This heightened identification naturally translates into affective commitment, as employees who strongly identify with their organization are more likely to develop an emotional attachment to it. This attachment is not merely emotional but also includes a cognitive alignment with the organization’s mission and vision. As a result, employees who identify strongly with their organization are more likely to also feel a sense of loyalty and duty, which are core components of normative commitment (El-Kassar et al., 2017).

The findings also revealed that HPHRPs are directly related to continuance commitment, and that organizational identification did not play the expected mediating role in this relationship. As proposed by SET (Blau, 1964), continuance commitment is primarily based on the perceived costs associated with leaving the organization. HPHRPs may be associated with job security, compensation, and benefits, thereby increasing the perceived costs of leaving directly, rather than through increased organizational identification.

The findings also elucidate the influential role of organizational culture as a moderator within the dynamic interplay between HPHRPs and organizational identification. SIT suggests that the alignment of individual and organizational values fosters identification. Organizational culture, which encompasses shared values and norms, intensifies this alignment. Tompkins and Cheney (1985) emphasize that a well-cultivated culture acts as a conduit for organizational control and identification. The nuanced influence of organizational culture can either strengthen or weaken organizational identification. In an emotionally neglectful culture, characterized by conventional thinking, low productivity, and burnout, the bond between the individual and the organization weakens. Conversely, a culture that values employee engagement is associated with increased participation, which relates to a stronger sense of identification.

Organizational culture also plays a positive moderating role between HPHRPs and both affective commitment and normative commitment, via organizational identification. When HPHRPs are implemented within a supportive and cohesive organizational culture, employees are more likely to perceive these practices as sincere efforts by the organization to invest in their well-being and development. This perception fosters a sense of belonging and emotional attachment, thereby improving affective commitment. Organizational culture also plays a critical role in shaping the link between HPHRPs, identification and normative commitment. In cultures that emphasize loyalty and long-term employment relationships, HPHRPs that promote stability and organizational support are likely to be interpreted as reinforcing these cultural values. Employees in such environments are likely to identify more with the organization and may feel a stronger moral obligation to remain with the organization due to the alignment between personal and organizational values.

## 6 Practical implications

These findings offer significant implications for managers seeking to foster employee commitment through the strategic use of HPHRPs. Given the positive relationship between HPHRPs and employee commitment, with organizational identification acting as a key mediating factor, managers should prioritize the implementation of HR practices that not only drive performance but also strengthen employees’ sense of belonging and identification with the organization. This can be achieved by fostering an environment where HR practices, such as training, performance appraisals, and rewards, are aligned with the organization’s core values and mission, thereby reinforcing employees’ connection to the organization (Era, 2024).

Moreover, managers must recognize the critical role of organizational culture as a moderating variable in this relationship. Organizational culture can amplify the positive relationship between HPHRPs and organizational identification and, subsequently, employee commitment. Managers should therefore focus on cultivating a supportive and cohesive organizational culture that reinforces the positive effects of HR practices. This includes promoting open communication, encouraging participation, and ensuring that the organization’s values are consistently demonstrated by leadership. By aligning HR practices with a positive organizational culture, managers can help foster employees’ sense of belonging and commitment.

However, managers must also be aware that the relationship between HPHRPs and different dimensions of employee commitment can vary. For instance, while organizational identification significantly mediates the relationship between HPHRPs and both affective and normative commitment, it may not play the same role in influencing continuance commitment. This indicates that managers should tailor their HR strategies to address the specific types of commitment they wish to foster. For example, while investments in training and development may be associated with higher affective commitment through increased identification, ensuring job security and career development opportunities may be more effective in bolstering continuance commitment.

## 7 Limitations and future research directions

The first limitation of this study is that data on HPHRPs, organizational identification, employee commitment and organizational culture were collected from a single source. Using data from the same participant can lead to common method biases (Gerhart et al., 2000), potentially overstating the correlation between variables and affecting the accuracy of the relationships in the study (Jakobsen and Jensen, 2015). Some respondents might exaggerate their commitment due to social desirability or prevailing trends. If employees evaluate themselves in an overly positive manner, a spurious positive correlation between the measured independent and dependent variables might occur, leading to biased results. Secondly, the cross-sectional methodology of this study limits the ability to determine causal relationships. Variables like organizational identification are dynamic; however, since data was collected at a single point in time, the study cannot account for how relationships between variables evolve over time (Ashforth et al., 2008). The dynamic nature of these relationships can be more deeply understood through longitudinal research in the future. The third limitation is the study’s focus on organizational identification while overlooking other important mechanisms that influence employee commitment. Although organizational identification is considered a mediator between HPHRPs and employee commitment, other potential mediators such as job satisfaction and perceived organizational support also play significant roles in this relationship (Iqbal et al., 2020; Nisar et al., 2017; Mahmood et al., 2019). Finally, this study only used data from Chinese organizations, which poses questions about the applicability of the findings across different cultural contexts. According to Su and Wright (2012), employees in Eastern and Western cultures may respond differently to HPHRPs, suggesting that the universality of findings across cultures merits further exploration.

## 8 Conclusion

In conclusion, the framework of this study lays a foundation for understanding the complex relationships among HPHRPs, organizational culture, organizational identification, and employee commitment. Future research should pursue longitudinal designs to track changes over time, uncover trends and potential causal relationships while minimizing respondent bias. Additionally, cross-cultural comparisons could reveal how cultural differences could impact these dynamics.

## Data Availability

The raw data supporting the conclusions of this article will be made available by the authors, without undue reservation.
